# Assisting hand function after spinal cord injury with a fabric-based soft robotic glove

**DOI:** 10.1186/s12984-018-0391-x

**Published:** 2018-06-28

**Authors:** Leonardo Cappello, Jan T. Meyer, Kevin C. Galloway, Jeffrey D. Peisner, Rachael Granberry, Diana A. Wagner, Sven Engelhardt, Sabrina Paganoni, Conor J. Walsh

**Affiliations:** 1 John A. Paulson School of Engineering and Applied Sciences, Harvard University, Pierce Hall, 29 Oxford Street, Cambridge, MA 02138 USA; 2 Wyss Institute for Biologically Inspired Engineering, Harvard University, 60 Oxford Street, Cambridge, MA 02138 USA; 3 Department of Physical Medicine and Rehabilitation, Harvard Medical School, Spaulding Rehabilitation Hospital, 300 First Ave, Boston, MA 02129 USA

**Keywords:** Soft robotic glove, Fabric-based robot, Spinal cord injury, Assistive wearable robot, Activities of daily living

## Abstract

**Background:**

Spinal cord injury is a devastating condition that can dramatically impact hand motor function. Passive and active assistive devices are becoming more commonly used to enhance lost hand strength and dexterity. Soft robotics is an emerging discipline that combines the classical principles of robotics with soft materials and could provide a new class of active assistive devices. Soft robotic assistive devices enable a human-robot interaction facilitated by compliant and light-weight structures. The scope of this work was to demonstrate that a fabric-based soft robotic glove can effectively assist participants affected by spinal cord injury in manipulating objects encountered in daily living.

**Methods:**

The Toronto Rehabilitation Institute Hand Function Test was administered to 9 participants with C4-C7 spinal cord injuries to assess the functionality of the soft robotic glove. The test included object manipulation tasks commonly encountered during activities of daily living (ADL) and lift force measurements. The test was administered to each participant twice; once without the assistive glove to provide baseline data and once while wearing the assistive glove. The object manipulation subtests were evaluated using a linear mixed model, including interaction effects of variables such as time since injury. The lift force measures were separately evaluated using the Wilcoxon signed-rank test.

**Results:**

The soft robotic glove improved object manipulation in ADL tasks. The difference in mean scores between baseline and assisted conditions was significant across all participants and for all manipulated objects. An improvement of 33.42 ± 15.43% relative to the maximal test score indicates that the glove sufficiently enhances hand function during ADL tasks. Moreover, lift force also increased when using the assistive soft robotic glove, further demonstrating the effectiveness of the device in assisting hand function.

**Conclusions:**

The results gathered in this study validate our fabric-based soft robotic glove as an effective device to assist hand function in individuals who have suffered upper limb paralysis following a spinal cord injury.

**Electronic supplementary material:**

The online version of this article (10.1186/s12984-018-0391-x) contains supplementary material, which is available to authorized users.

## Background

Each year, approximately 12,500 Americans survive spinal cord injuries (SCI) [1] and in 2016 the population of SCI was estimated to be 276,000 individuals, 906 per million people [2]. A major and devastating result of cervical-level SCI is the drastic reduction of upper extremity function, specifically the hands, a condition which can impact independence and quality of life of those affected. Incomplete tetraplegia is the most frequent neurologic deficit at discharge (45%) [1] and Anderson et al. found that 48.7% of surveyed tetraplegics indicated that regaining arm and hand function would have the greatest impact to improve their quality of life [3]. While recovery of hand function is highly desired after SCI, limited treatments are available to aid in recovery despite the increasing knowledge of this medical condition [4].

Many individuals with hand paralysis who retain wrist motor function can generate a type of passive grasp called tenodesis grasp. This passive grasp relies on the weak elasticity of the hand muscle fibers and of the connective tissue elements composing the muscle-tendon-bone unit. Tenodesis grasp functions by contracting extensor muscles in the wrist and forearm through wrist extension. This action pulls the finger tendons towards the wrist, forcing a bending moment, which can be employed to produce a grasping posture in the paralyzed hand. The passive forces produced by a tenodesis grasp are weak and generally only sufficient to lift lightweight objects when the forearm is supinated and gravity is assisting [5]. An active grasp would therefore benefit SCI patients by allowing them to lift heavier objects and manipulate them in free space.

Robotics is currently proposed as a non-invasive solution to enhance hand functionality by means of wearable, actuated platforms, namely robotic exoskeletons, which are able to move the hands of the impaired users and assist functionality. A number of robotic exoskeletons for the distal upper limb have been designed over the past years, which can be classified based on their actuation principles, materials employed, complexity, and integrated functions. Many of these devices rely on rigid linkages, which require careful alignment with the human joints to safely and effectively transfer their robust and reliable forces and torques to the wearer. Rigid exoskeletons are well-suited for challenging clinical scenarios, e.g., rehabilitation of the wrist [6, 7], the hand [8, 9], and the individual fingers [10] (for exhaustive reviews see [11, 12]), however the design trade-off for high forces that characterize these rigid mechanical designs is their limited portability due to the weight of the electromechanical actuators and their rigid frames. Consequently, most of the existing training systems are stationary, designed for in-clinic use, and require experienced personnel to oversee the patient’s safety and wellbeing during usage.

Compliant, lightweight, and mobile devices designed for home-use provide a new paradigm of assistive devices. Cable-driven transmissions enable reduced weight and increased compliance of wearable, robotic systems. This approach relieves the distal extremities from heavy actuation units and electronics by relocating them to more convenient locations, retaining rigid frames [13–17] or replacing them with polymer-based [18] or fabric-based [19–24] structures, thereby increasing the portability, comfort, and usability of the devices. While cable-driven systems have been developed to improve usability, reduce weight, and maximize compliance, the inevitable tradeoff is diminished strength and accuracy compared to traditional, rigid exoskeletons. A limited number of research groups successfully demonstrated cable-driven wearable robots to assist hand function in SCI survivors [16, 17]. Additionally, elastomers [25–32] and more recently textiles [33–35] have been employed instead of traditional, rigid actuators and have been shown to offer a new class of soft wearable robotic devices. These recent efforts offer the potential for safe, comfortable, lightweight and cost-effective devices that can provide users with at-home assistance or rehabilitation activities for prolonged periods [25–27].

The aim of this work is to demonstrate that a fabric-based soft robotic glove, which is portable, bidirectional (i.e. assists both hand opening and closing), and multi-posture (i.e. enables palmar grasp and pinch grasp) offers a viable assistive solution for participants with limited hand strength and dexterity. To assess the effectiveness of the assistive glove, we administered a clinical motor function test to evaluate the grasping function of untrained participants with impaired hand motor function due to SCI with and without assistance provided by a soft robotic glove. Although voluntary control is a crucial aspect when evaluating the feasibility of an assistive device, this work focused only on the mechanical performance of the proposed soft robotic glove to decouple functionality from control logistics.

## Methods

### Fabric-based soft robotic glove

**Fig. 1:**
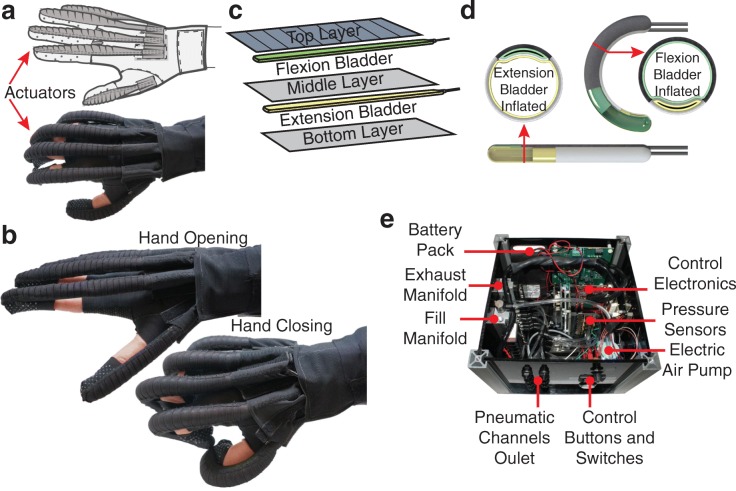
Soft robotic glove and control box characteristics: **a** Schematic of the soft robotic glove **b** Basic motion principle of the glove **c** Construction approach with different fabric layers of specific material properties and enclosed air bladders **d** Operating principle: finger extension and flexion motion are obtained when the respective chambers are pressurized **e** Portable, self-contained box used to actuate and control the glove

Prior iterations of our soft robotic glove [25–27] incorporated unidirectional molded elastomeric actuators. Figure 1a-b depicts a more recent model of the glove designed with bidirectional, fabric-based actuators, which are lighter than their elastomeric predecessors (see Additional file 1: Figure S1). These fabric-based soft robotic actuators are described in detail in [35]. Our updated design is composed of a base glove with attachments points (i.e. hook and loop fasteners, straps) for modular, independent finger actuators. Each fabric-based actuator is comprised of three fabric layers and two air-tight bladders placed between each fabric pocket. Finger flexion and extension is obtained by selectively pressurizing these bladders with an air pump. By leveraging the material properties of each fabric layer (e.g. stiffness, anisotropy) and by incorporating geometric design variables into the structure (i.e. pleats), the actuators are designed to accomplish complex motions that mimic the natural movements of the hand [35].

The use of fabric and other compliant materials in a soft robotic glove allows our device to resemble the dimensions and weight of a common padded wheelchair glove. The inclusion of fabric-based, soft robotic actuators into the design resulted in a device that is significantly lighter than our past designs (77 g here vs. 285 g in [26]). To optimize fit and improve performance, a modular sizing system has been adopted: four base glove sizes (small, medium, large and extra-large) were designed to provide a base for attachable actuation modules. Three different size actuators (small, medium, and large), each with two different size finger pockets (narrow and wide) could be easily mounted to the base glove through a combination of hook and loop fastener and straps. This semi-customizable approach (4 base gloves × 3 actuators × 2 pockets  × 5 fingers = 120 possible combinations) allowed each participant to find the best fit based on the unique size of the individual’s hand and fingers. This sizing scheme was based on measurements from the hands of healthy individuals (12 females and 20 males). During this measurement study, we collected over 50 dimensions in multiple working postures, including circumferences of the palmar region and fingers as well as the lengths of each phalange. Moreover, to maximize grip, the palmar surface of the glove featured silicon ripples.

The soft robotic glove is designed to apply sufficient force to open and close each finger, which enables grasping and lifting of light objects. Due to its intrinsically compliant structure, the glove is capable of grasping objects with different shapes. It was previously demonstrated that the glove can make a healthy user grasp an object with a force of 15 N corresponding to an average of approximately 30% of the maximum pinch force of a healthy adult [36]. It was calculated as the integral of contact pressure measured with an ultra-thin pressure mapping sheet (5250, TekScan Inc., USA) wrapped around a cylinder with diameter of 76 mm [35] while the user kept the muscles passive and the glove was pressurized to 172 kPa (25 psi), as determined by the specific electric air pump used (D1011–23-01, Parker Hannifin, USA).

To control the glove, a portable and self-contained control box (Fig. 1e-f) was developed. The box comprises: i) the control electronics, ii) a battery pack, iii) the electric air pump, iv) an exhaust and fill air manifold where each of v) seven solenoid valves were connected to control the airflow of vi) seven pneumatic channels, ultimately connected to the pneumatic actuators. A pressure control loop was implemented to switch on the electric pump and to drive the solenoid valves. The single finger actuators are either controlled individually, or paired to joint pneumatic channels, to reduce complexity. The seven pneumatic channels included: i) thumb flexion, ii) thumb extension, iii) index finger flexion, iv) paired index and middle finger extension, v) middle finger flexion, vi) paired pinky and ring finger extension, and vii) paired pinky and ring finger flexion. The dimensions and weight of the box (25 × 25 × 20 cm, 5 kg) were purposely kept as low as possible for easy portability, e.g. mounted on a wheelchair, or placed on a table.

The glove performs a 3-point pinch grasp when thumb, index, and middle finger flexion are individually actuated. When all flexion actuators - including pinky and ring finger - are triggered, the glove performs a palmar grasp. During this study, the control box was operated by a member of the research team who could select the grip type among the two available options with a rotary switch. Two buttons triggered hand opening (finger extension) and closing (finger flexion) motions by alternatively pressurizing the agonist actuators while depressurizing the antagonist ones. A third button was included to discharge the residual air in the bladders when no active assistance was needed and as a safety feature. The glove was controlled by the researcher after verbally confirming each intended motion with the study participant.

### Participants

Nine participants (8 males, 1 female, age range 20–68 years, see Table 1) were enrolled in this study, which was performed in accordance with the Declaration of Helsinki and approved by the Harvard Medical School Institutional Review Board. Eligible participants fulfilled the following inclusion criteria: i) age between 18 and 70 years, ii) diagnosed with C4-C7 spinal cord injury, iii) loss of hand function, specifically strength and/or range of motion, iv) understanding and speaking English as well as a score ≥ 23 on the Mini Mental State Examination (MMSE) administered by the researchers prior to the study session. All the admitted participants had a high level of general disability (tetraplegia) and were wheelchair-bound. All participants gave their written informed consent either themselves or by their legal guardian. Participants were recruited through rehabilitation clinics in the Greater Boston Area and screened during a first visit, in which they were instructed on the testing procedure and familiarized with the robotic device.

**Table 1:** Demographic data of the participants enrolled in the pilot study

	Age	Gender	Level of Spinal Injury	Time Since Injury
Participant 1 (P1)	68	M	C7	44 years
Participant 2 (P2)	20	M	C5	1 year
Participant 3 (P3)	49	F	C5-C6	33 years
Participant 4 (P4)	65	M	C6	44 years
Participant 5 (P5)	45	M	C5-C6	25 years
Participant 6 (P6)	63	M	C5	50 years
Participant 7 (P7)	56	M	C4-C5	38 years
Participant 8 (P8)	53	M	C7	5 months
Participant 9 (P9)	30	M	C5-C7	7 years

### Experimental conditions

The study followed a case series design, where each participant was asked to perform the baseline condition first, followed by the assisted condition wearing the soft robotic glove. The baseline condition consisted of executing a clinically relevant test to assess the gross motor function of the impaired hand without assistance. During the test, participants were not allowed to wear passive devices, including those normally worn by some of the participants, such as splints or padded gloves to avoid unpredictable effects that these devices could have on the bare hand function. This test was followed by an assisted condition conducted with the participants wearing the soft robotic glove in the active state. The assistive glove was used on the preferred hand of each participant. In both conditions, the participants were allowed to use the contralateral hand to place objects in the studied hand and to stabilize the initial grasp. Object lifting and manipulation was then executed only with the selected hand under investigation. Given the full portability of the system, the study sessions were carried either in the Harvard Biodesign Laboratory, Spaulding Rehabilitation Hospital Cambridge, or participant’s homes depending on each participants’ personal availability.

**Fig. 2:**
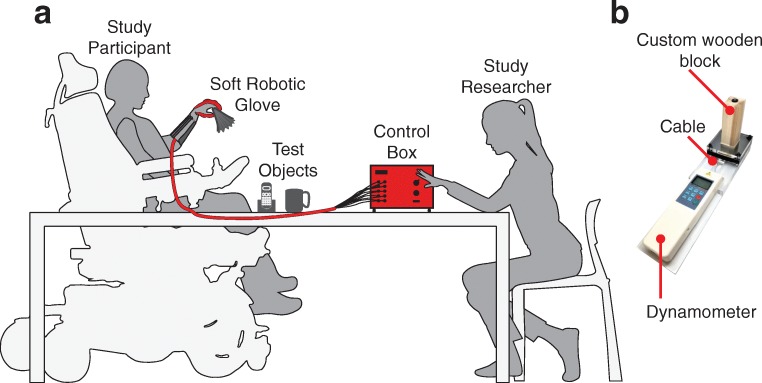
The experimental setup: **a** The study procedure was conducted on a regular table, with the participant approaching and positioning themselves as preferred and as their individual wheelchair permitted. The TRI-HFT test objects, as well as the control box, operated by the study researcher, were placed on the table in front of the study participant. **b** The custom-made wooden block connected to a dynamometer through a cable to measure lift force

We selected the Toronto Rehabilitation Institute Hand Function Test (TRI-HFT) [37] as the outcome measure for this study because it was specifically designed to measure unilateral gross motor function during palmar grasp, lateral pinch and pulp pinch in persons with SCI. The TRI-HFT has been previously used to measure the effect of a training program for restoring upper extremity reaching function using a neuroprosthesis [38] as well as to measure the effect of a hand assistive device to restore autonomy in participants with SCI [16]. The TRI-HFT assesses a person’s ability to manipulate objects and weights that would be encountered while performing ADL. Additionally, it assesses the forces that can be produced by the hand. The test can be divided into three parts: the first section consists of an object manipulation test (10 ADL objects, max. 7 points per object) that requires two main grasp types, namely the palmar grasp (objects 1,3,5,7,9) and pinch grasp (objects 2,4,6,8,10). In the second part - the wooden block strength test - the participant is asked to manipulate wooden blocks of various masses and surface finishes (objects 11–19, max. 7 points per object). The final part consists of a series of dynamometric measures to quantitatively assess the lift forces produced by the participants. As such, this section of the TRI-HFT is rated in Newtons and is not validated [37], representing a secondary outcome measure. The instruments to measure lift force were built in our laboratory. In this section, a dynamometer is connected to a cylinder, a credit card (as described in [37]), and a custom-made wooden block similar to object 12 (40 × 40 × 120 mm, 100 g) to measure the maximum lift force produced with both a palmar grasp and a pinch grasp (Fig. 2b). We decided to include this item in the original test to gather lift force data on the power and pinch grasps, and thus perform more measures to capture differences in dynamometric scores between the two conditions. The TRI-HFT primary outcome consists of a maximum potential score of 133 points through the combination of 19 object manipulations, and the secondary outcome consists of the dynamometric measures.

The experiments were conducted with the test objects and the control box placed on a regular table, which the participants approached as close as their wheelchair allowed. The control box was operated by the researcher sitting next to the participant (Fig. 2). The presentation order of the tasks and objects was performed according to the original method proposed by Kapadia et al. [37], starting with the object manipulation test, followed by the wooden block strength test and lastly dynamometric measurements. The baseline evaluation was always conducted first, followed by the assisted session. While the baseline test was being conducted, at least one researcher assembled the assistive glove to meet the participants’ hand specifications. This sequence reduced the overall time and workload of the study session. Sufficient breaks were included throughout the administration of the test to allow the participants to rest and thereby minimize muscle fatigue effects. There were no time restrictions on duration of task performance and the participants could indicate the end of the task if they became fatigued or judged the task too difficult to accomplish.

### Data collection

All TRI-HFT trials were recorded on video for rating post-test. Due to high interrater reliability (ICC = 0.98, [37]), two independent raters were chosen to evaluate each participant based on the guidelines of the initial study by Kapadia et al. in 2012. Any discrepancies between the two raters’ scores were resolved by discussion to achieve a consensus, with input if necessary from a third independent researcher oriented to the TRI-HFT score criteria. Dynamometric measurements were recorded with a handheld force gauge (HF-50, Shenzen Tony Electronics, China) capable of storing the maximum force measured during the trial. The custom-made block and supporting platform was devised to only permit and measure the application of upward vertical forces. The other TRI-HFT dynamometric assessments were performed strictly following the visual instructions of [37] with consistent speed across the participants to minimize the influence of direction of force and pulling speed in the outcome measures.

### Statistical analysis

TRI-HFT scores of the object manipulation, wooden block test as well as the dynamometric measurements were evaluated for all participants. A linear mixed model was fitted to evaluate the difference in the TRI-HFT scores between the baseline and assisted condition. The random effects tested for possible interactions with the model included participants, objects, and time since injury. The force measurements were evaluated with a Wilcoxon signed-rank test to test for significance in lift force difference. The data were processed and analyzed with Matlab (MathWorks, Natick, USA) using the “fitlme” class tools as well as the “signrank” function.

## Results

**Fig. 3:**
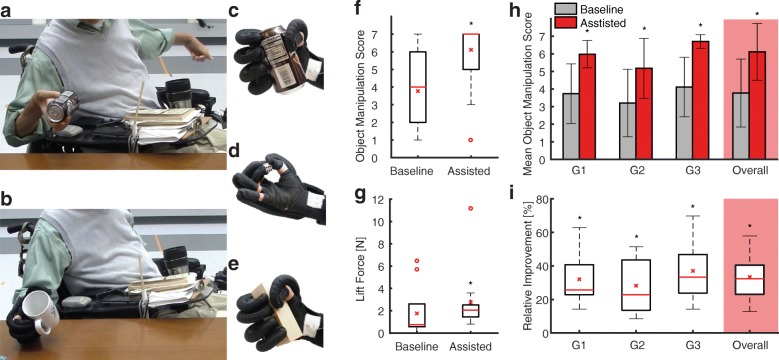
Soft robotic glove for hand function restoration: **a** Study participant during unassisted object manipulation using a passive tenodesis grasp to lift an object (baseline condition) **b** Study participant performing an active palmar grasp to manipulate an object using the soft robotic glove (assisted condition). **c** Soft robotic glove assisting palmar grasp **d** Soft robotic glove assisting pinch grasp **e**. Soft robotic glove assisting the grasp of a wooden block to assess lift force **f** Improvement in TRI-HFT score across all participants (*N* = 9; baseline mean = 3.77, median = 4; assisted mean = 6.11, median = 7; mean difference = 2.34; 95% confidence interval from 1.67 to 3.01; *p* < 3e-11) **g** Improvement in mean lift force across all participants (N = 9; baseline mean = 1.76 N, median = 0.68 N; assisted mean = 2.76 N, median = 2.03 N; mean difference = 1.00 N; Wilcoxon signed-rank test, Z = − 4.28, *p* < 2e-5) **h** Mean object manipulation scores of palmar grasp (G1), pinch grasp (G2) and wooden block strength (G3) subtests. The mean score difference between the baseline and assisted condition is significant and consistent throughout all subgroups (see 2F). The average scores between the groups differ significantly (Linear Mixed Model Coefficient Test, G1/G2; F = 18.81, p < 2e-4, G2/G3; F = 10.63, *p* < 1.5e-3, G2/G3; F = 6.85 *p* < 0.01) **i** Subgroup specific improvement of the TRI-HFT score from baseline to the assisted condition, relative to the maximum value = 7 (G1 mean = 32.06%, median = 25.71%; G2 mean = 28.25%, median = 22.85%; G3 mean = 37.04%, median = 33.33%; overall mean = 33.42%, median = 31.85%). * indicates significant difference with respect to the baseline condition, red stars denote means, red lines denote medians, red circles denote outliers defined as data points outside the range of 1.5 times the interquartile range

The proposed soft robotic glove improved key hand functions to manipulate ADL objects (Fig. 3a-e). Long-term effects that may arise from sustained use of the device were not an object of this study. The difference in mean score between the baseline and assisted condition improved significantly across all subtests within the TRI-HFT (mean score difference = 2.34, 95% confidence interval from 1.67 to 3.01, *p* < 3e-11, Fig. 3f). Grouping objects by their corresponding grip types and subtests - Objects 1, 3, 5, 7, 10 representing a palmar grasp (G1), objects 2, 4, 6, 8, 9 involving pinch grasp (G2) and objects 11–19 for the wooden block strength measures (G3) - shows that the average score between palmar grasp, pinch grip and wooden block strength varies but the difference between the baseline and assisted condition remains constant (linear mixed model coefficient test, G1-G2; F = 18.81, *p* < 2e-4, G2-G3; F = 10.63, *p* < 1.5e-3, G2-G3; F = 6.85 *p* < 0.01, Fig. 3h). The soft robotic glove improved the mean TRI-HFT scores of the object manipulation tests overall by 33.42 ± 15.43% relative to the maximum achievable score (7 points) (Fig. 3i), from a mean of 53.88 ± 24.20% to a mean of 87.30 ± 11.82% of the total maximum score (133 points). Time since injury did not significantly influence the object manipulation performance (*p* > 0.11). It is worth noting that the assistance of the glove reduced the variability of the participants’ performance.

**Fig. 4:**
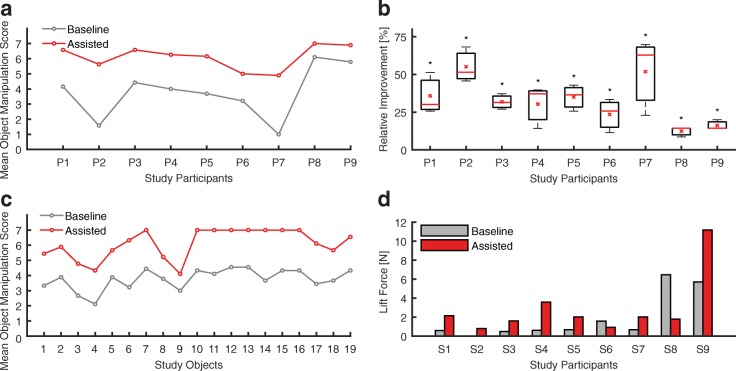
Individual participant and object TRI-HFT measures: **a**. Mean manipulation score across all subtests for each individual study participant. The mean difference between the assisted and baseline condition is significant and constant (mean difference = 2.34, p < 3e-11, see 3F) **b**. Object manipulation improvement of each study participant from baseline to assisted relative to the maximum score = 7 (S1 mean = 35.77%, median = 30.16%; S2 mean = 55.13%, median = 51.43%; S3 mean = 31.85%, median = 31.43%; S4 mean = 30.37%, median = 37.14%; S5 mean = 35.03%, median = 36.51%; S6 mean = 23.49%, median = 25.71%; S7 mean = 51.85%, median = 62.86%; S8 mean = 12.38%, median = 14.29%; S9 mean = 16.19%, median = 14.29%) **c**. Mean score across all participants for each individual study object, listed in presentation order. Objects 1, 3, 5, 7, 10 represent the palmar grasp, object 2, 4, 6, 8, 9 pinch grasp, and objects 11–19 composed the wooden block strength subtest. The mean difference between the assisted and baseline condition is significant and constant (mean difference = 2.34, p < 3e-11, see 3F) **d** Mean lift force across all force measurements per study participant. The overall mean difference across participants between the baseline and assisted condition is significant (Wilcoxon signed-rank test, Z = − 4.28, p < 2e-5). * indicates significant difference with respect to the baseline condition, red stars denote means, red lines denote medians

Individual improvements of all study participants are reported in Fig. 4a-b. The mean difference between the baseline and assisted condition is consistent across all participants and, therefore, generally unaffected by the difference in individual objects (Fig. 4c). No sign of muscular fatigue is interpretable from the performances of the participants within the test results (Fig. 4c).

As for the secondary outcome of the TRI-HFT, we focused on the overall improvement across all dynamometric measures of lift force rather than analyzing each test method and individual participant. A significant increase in lift force of 1.00 N (mean baseline force 1.76 ± 4.32 N, mean assisted force 2.76 ± 5.18 N) when using the assisting soft robotic glove is observable (Wilcoxon signed-rank test, Z = − 4.28, *p* < 2e-5, Fig. 3g). The individual participant’s lift forces represented in Fig. 4d help gaining insight on the outliers displayed in Fig. 3g**.** It is worth noting that the eccentric wooden bar was not used in this study because none of the study participants were capable of holding it.

## Discussion

The effect of active assistance from a fully portable, fabric-based soft robotic glove on hand function was evaluated in participants with SCI. The Toronto Rehabilitation Institute Hand Function Test (TRI-HFT) was used to assess the grasping and manipulation capabilities of the study participants in baseline (no glove) and active conditions (glove powered).

With the glove actively supporting grip, participants on average performed 87.30 ± 11.82% on the TRI-HFT. Compared to the baseline average performance of 53.88 ± 24.20% when the participant was not wearing the glove, the improved score and the reduced variability in the assisted condition suggest the glove is capable of enhancing hand function of participants with disparate unaided performance to a similar, more functional level.

Individuals with low baseline scores (i.e. participants 2 and 7 with C4-C5 level of injury) particularly benefitted from the soft robotic glove, with an improvement in the primary outcome of the TRI-HFT of over 50%. In addition, participants 8 and 9 with SCI of lower lesion levels (C6-C7) scored high in the baseline condition but still benefitted from the assistance provided by the glove. It is worth noting that the large standard deviation characterizing the baseline condition is reduced in the assisted condition, demonstrating that the glove provided a reliable and consistent assistance across all subtests.

The glove provided the most consistent assistance during the wooden block strength section, likely due to the consistent block size and shape. The difference in surface textures of the blocks did not significantly impact the performance, however the increasing weight did impact the participants’ ability to lift.

We observed that the glove did not perform an optimal pinch grasp with specific objects like the Ziploc bag filled with golf balls (Object 4) or the pencil (Object 9). Due to the current actuator design and glove architecture: a powerful contact between the thumb and index finger is challenging. Generally, we can conclude that the glove provided a very firm and reliable palmar grasp, however the pinch grasp needs further improvement and investigation.

In addition to demonstrating improved object manipulation, we also saw positive effects in the dynamometric measures. Wearing the glove was shown to enable a firmer grip compared to the bare hand: the significant increase of lift force of 1 N allows the users to lift a larger number of objects towards increased independency. Based on the list of objects of daily living compiled by Matheus and Dollar [39], the glove enables an average impaired user to lift about 59% of the objects of daily living, 11% more than the baseline, and would return the capability of lifting about 33% of these objects to a user with no hand function. In participants 6 and 8, we can observe a reduction in lift force from baseline to assisted condition that may be due to the combination of two factors: i) the stiffness of their muscles and ii) the passive execution of the assisted condition. Participants 6 and 8, in fact, performed a firm tenodesis grasp probably due to the large muscle stiffness, which produced large lift forces but resisted the effect of the glove. Furthermore, we hypothesize that participants 6 and 8, did not actively support the action of the glove with tenodesis in this section of the test, thus resulting in lower forces compared to the baseline. It is worth noting that the action of the glove was enough to allow these two participants to obtain a higher score in the other sections of the TRI-HFT compared to the baseline condition, suggesting that the glove does not negatively affect grasping.

Although no structured comfort or usability questionnaires were completed during this study, no inherent discomfort was reported from using the soft robotic glove or the associated instrumentation. Additionally, every participant stated that they would benefit from a daily domestic use of the glove to perform ADL independently. The participants also noted that they would be open to wearing the glove all day due to its light weight and minimal obtrusiveness. Participants 8 and 9, who were capable of performing a firm passive tenodesis grasp during baseline testing, reported that the glove allowed them to rely on its assistance and thereby minimized any required wrist flexion to passively trigger hand closing posture. Most of the participants reported that they would benefit from a lighter and more compact control box to be placed on the wheelchair and some of them reported that the air pump was too noisy. Finally, the participants were unable to independently don the glove and suggested this feature in the next generation device.

The presented glove differs from other devices due to its lightweight and compliant design, however the TRI-HFT results showed comparable improvements with respect to [16], where an assistive cable-driven robotic glove was presented and tested in six SCI participants. In [17], another group showed that their cable-driven robotic device successfully assisted hand function in two SCI participants, assessed using the Sollerman Hand Function Test (SHIFT). In a further study [23], the authors employ a soft wearable glove to assist ADL in 28 elderly people. As evaluated by the System Usability Scale, the study showed that these users are likely to accept a soft wearable glove for daily use. These exciting recent studies in SCI and elderly populations demonstrate the potential for lightweight assistive devices such as the proposed soft robotic glove to restore hand function with small levels of assistance. Many other soft wearable robots have been devised to assist hand function, either based on cable transmission [16–18, 20, 21] or pneumatic actuation [25–28, 30, 33, 35]; however, these efforts focused on the design and development of the devices rather than validation.

In the field of rehabilitation robotics, many rigid platforms [6–8, 10, 11] or wearable gloves [9, 13, 15, 24, 29, 31, 33] have been specifically designed to deliver neuromuscular therapy and focused their assessment on longitudinal performance measures. While the robotic platform proposed in this paper was not intended for rehabilitation, we believe that it could potentially be deployed with minimal design changes to deliver physical therapy either as an active robotic trainer or as a continuous passive motion device, similar to commercially available devices (Hand Mentor Pro, Motus Nova, USA). Consequently, future studies will focus on developing the soft robotic glove as a multi-purpose platform for scenarios where both manipulation assistance and high-dose physical therapy treatments are desirable.

Based on the results and the observations obtained in this study, we believe that the performance of our fabric-based soft robotic glove could be further improved. For some of the older participants, the assistive glove negated decades of learned motor behaviors and we believe that the performance may improve if participants had more time wearing the glove outside of the study, which might enable them to develop innovative strategies of completing tasks in their environment. Furthermore, the pinch grasp can be enhanced by designing the actuators to be more conformal to the anatomy of the human finger, allowing the forces generated by the device to be adequately transferred to the hand. Additionally, in future work pressure and strain sensors will be integrated into the palmar surface of the glove and into the fingers, respectively to measure contact force and bending status of the actuators. This will offer grip strength and grasp posture feedback that can be fed to a closed-loop controller as well as produce trigger signals to control the glove. Moreover, the portable control box dimensions will be optimized to enable in-home everyday usage, the possibility for individuals with limited hand function to don and doff the glove autonomously will be included for independent use, and we will evaluate replacing the air pump with a compressed air cartridge to reduce noise. Finally, the aforementioned closed-loop controller is under development that will support a portable integrated platform capable of detecting a user’s intentions and delivering appropriate and natural assistance accordingly. Prior experience of the research team in using electromyography (EMG) to control soft pneumatic gloves [25] will be leveraged towards the implementation of a responsive device based on the voluntary activation of intact muscle groups. Future research will need to provide evidence of robustness and controllability of the glove, perform intensive testing sessions of at-home usage, administer usability questionnaires, and gather users’ suggestions for further development through focus groups or other means. Despite the encouraging results, this work represents just the first step of the holistic approach required to assess whether an assistive device can be adopted and integrated into the end-user’s daily routine.

## Conclusions

This study provides insight on the viability of a fabric-based soft robotic glove to restore basic hand function. The administration of the TRI-HFT to individuals who suffered upper limb paralysis after spinal cord injury showed a significant average object manipulation improvement of 33.42% relative to the maximum score as well as significant average improvement of the maximum achievable lift force. Being fully portable, the proposed soft robotic glove is versatile enough to be used both in clinical as well as home settings. Moreover, its restoring effects on manipulation ability are comparable to those produced by similar systems found in literature. In summary, this pilot study paves the way for a fabric-based soft robotic glove to enhance dexterity and strength in ADL in people who suffered hand paralysis.

## Additional file

Additional file 1: Additional views of the fabric-based soft robotic glove. (PDF 430 kb)

## Abbreviations

ADL: Activities of daily living
ICC: Intraclass correlation coefficient
MMSE: Mini mental state exam
SCI: Spinal cord injury
TRI-HFT: Toronto rehabilitation institute hand function test

## References

[1] National Spinal Cord Injury Statistical Center (2016). Spinal cord injury. Facts and figures at a glance. J Spinal Cord Med.

[2] Richards C, Mackenzie N, Roberts S, Escorpizo R (2017). People with Spinal Cord Injury in the United States. Am J Phys Med Rehabil.

[3] Anderson KD (2004). Targeting recovery: priorities of the spinal cord-injured population. J Neurotrauma.

[4] Hachem LD, Ahuja CS, Fehlings MG (2017). Assessment and management of acute spinal cord injury: from point of injury to rehabilitation. J Spinal Cord Med.

[5] Johanson ME, Murray WM (2002). The unoperated hand: the role of passive forces in hand function after tetraplegia. Hand Clin.

[6] Krebs HI, Volpe BT, Williams D, Celestino J, Charles SK, Lynch D, Hogan N (2007). Robot-aided neurorehabilitation: a robot for wrist rehabilitation. IEEE Trans Neural Syst Rehabil Eng..

[7] Pehlivan AU, Sergi F, Erwin A, Yozbatiran N, Francisco GE, O’Malley MK. Design and validation of the RiceWrist-S exoskeleton for robotic rehabilitation after incomplete spinal cord injury. Robotica. 2014:1–17. 10.1017/S0263574714001490.

[8] Schabowsky CN, Godfrey SB, Holley RJ, Lum PS (2010). Development and pilot testing of HEXORR: hand EXOskeleton rehabilitation robot. J Neuroeng Rehabil..

[9] Ates S, Haarman CJW, Stienen AHA. SCRIPT passive orthosis: design of interactive hand and wrist exoskeleton for rehabilitation at home after stroke. Auton Robots. 2016; 10.1007/s10514-016-9589-6.

[10] Polotto A, Modulo F, Flumian F, Xiao ZG, Boscariol P, Menon C (2012). Index finger rehabilitation/assistive device. Proc - IEEE RAS EMBS Int Conf Biomed Robot Biomechatron.

[11] Riener R, Nef T, Colombo G (2005). Robot-aided neurorehabilitation of the upper extremities. Med Biol Eng Comput.

[12] Maciejasz P, Eschweiler J, Gerlach-Hahn K, Jansen-Troy A, Leonhardt S (2014). A survey on robotic devices for upper limb rehabilitation. J Neuroeng Rehabil.

[13] Chiri A, Vitiello N, Giovacchini F, Roccella S, Vecchi F, Carrozza MC (2012). Mechatronic design and characterization of the index finger module of a hand exoskeleton for post-stroke rehabilitation. IEEE/ASME Trans Mechatronics.

[14] Jones CL, Wang F, Morrison R, Sarkar N, Kamper DG (2014). Design and development of the cable actuated finger exoskeleton for hand rehabilitation following stroke. IEEE/ASME Trans Mechatronics..

[15] Mao Y, Agrawal SK. Design of a Cable-Driven Arm Exoskeleton (CAREX) for Neural Rehabilitation. Trans Robot. 2012;28:922–31.

[16] Soekadar SR, Witkowski M, Gómez C, Opisso E, Medina J, Cortese M, Cempini M, Carrozza MC, Cohen LG, Birbaumer N, Vitiello N. Hybrid EEG/EOG-based brain/neural hand exoskeleton restores fully independent daily living activities after quadriplegia. Sci Robot. 2016;1:eaag3296. 10.1126/scirobotics.aag3296. 10.1126/scirobotics.aag3296 33157855

[17] Yun Y, Dancausse S, Esmatloo P, Serrato A, Merring CA, Agarwal P, Deshpande AD. Maestro: an EMG-driven assistive hand exoskeleton for spinal cord injury patients. Proc - IEEE Int Conf Robot Autom. 2017:2904–10.

[18] Kang BB, Lee H, In H, Jeong U, Chung J, Cho KJ (2016). Development of a polymer-based tendon-driven wearable robotic hand. Proc - IEEE Int Conf Robot and Autom.

[19] Galiana I, Hammond FL, Howe RD, Popovic MB (2012). Wearable soft robotic device for post-stroke shoulder rehabilitation: identifying misalignments. Proc - IEEE/RSJ Int Conf Int Robot Syst (IROS).

[20] Xiloyannis M, Cappello L, Dinh BK, Antuvan CW, Masia L (2017). Design and preliminary testing of a soft exosuit for assisting elbow movements and hand grasping. Biosystems and biorobotics.

[21] Cappello L, Binh DK, Yen SC, Masia L (2016). Design and preliminary characterization of a soft wearable exoskeleton for upper limb. Proc - IEEE RAS EMBS Int Conf Biomed Robot Biomechatron.

[22] Gaponov I, Popov D, Lee SJ, Ryu J (2016). Auxilio : a portable cable-driven exosuit for upper extremity assistance. Int J Control Autom Syst.

[23] Radder B, Prange-Lasonder GB, AIR K, Gaasbeek L, Holmberg J, Meyer T, Buurke JH, Rietman JS (2016). Preliminary findings of feasibility of a wearable soft-robotic glove supporting impaired hand function in daily life a soft-robotic glove supporting ADL of elderly people. Proc Int Conf Inf Commun Technol Ageing Well E-Health.

[24] Nilsson M, Ingvast J, Wikander J, Von Holst H. The Soft Extra Muscle system for improving the grasping capability in neurological rehabilitation. Proc - IEEE EMBS Conf Biomed Eng Sci (IECBES). 2012; December:412–417.

[25] Polygerinos P, Galloway KC, Sanan S, Herman M, Walsh CJ (2015). EMG controlled soft robotic glove for assistance during activities of daily living. Proc - IEEE Int Conf Rehab Robot (ICORR).

[26] Polygerinos P, Wang Z, Galloway KC, Wood RJ, Walsh CJ (2015). Soft robotic glove for combined assistance and at-home rehabilitation. Rob Auton Syst.

[27] Polygerinos P, Galloway KC, Savage E, Herman M, O’Donnell K, Walsh CJ. Soft robotic glove for hand rehabilitation and task specific training. Proc - IEEE Int Conf Robot Autom 2015; June:2913–2919.

[28] Zhao H, Jalving J, Huang R, Knepper R, Ruina A, Shepherd R (2016). A helping hand: soft orthosis with integrated optical strain sensors and EMG control. IEEE Robot Autom Mag.

[29] Connelly L, Jia Y, Toro ML, Stoykov ME, Kenyon RV, Kamper DG (2010). A pneumatic glove and immersive virtual reality environment for hand rehabilitative training after stroke. IEEE Trans Neural Syst Rehabil Eng.

[30] Yun S, Kang BB, Cho K. Exo-glove PM: an easily customizable modularized pneumatic assistive glove. IEEE Robot Autom Lett 2017;5 c:1. 10.1109/LRA.2017.2678545.

[31] Vargas PA, Brasil FL, McConnell AC, Vallejo M, Corne DW, Stokes AA, Moioli RC. Combining soft robotics and brain-machine interfaces for stroke rehabilitation. In: Ibáñez J., González-Vargas J., Azorín J., Akay M., Pons J. (eds) Converging Clinical and Engineering Research on Neurorehabilitation II. Biosystems & Biorobotics. Springer, Cham. 2017;15:1257-1262. 10.1007/978-3-319-46669-9_205.

[32] McConnell AC, Vallejo M, Moioli RC, Brasil FL, Secciani N, Nemitz MP, Riquart CP, Corne DW, Vargas PA, Stokes AA. SOPHIA: soft orthotic physiotherapy hand interactive aid. Front Mech Eng 2017;3 June:1–13. 10.3389/fmech.2017.00003.

[33] Yap HK, Khin PM, Koh TH, Sun Y, Liang X, Lim JH, Yeow CH (2017). A fully fabric-based bidirectional soft robotic glove for assistance and rehabilitation of hand impaired patients. IEEE Robot Autom Lett..

[34] Yap HK, Lim JH, Nasrallah F, Yeow C, Winslow B. Design and Preliminary Feasibility Study of a Soft Robotic Glove for Hand Function Assistance in Stroke Survivors. Front Neurosci. 2017;11 October:1–14. 10.3389/fnins.2017.00547 PMC5640819 29062267

[35] Cappello L, Galloway KC, Sanan S, Wagner DA, Granberry R, Engelhardt S, Haufe FL, Peisner JD, Walsh CJ. Exploiting textile mechanical anisotropy for fabric-based pneumatic actuators. Soft Robotics. 2018. 10.1089/soro.2017.0076. 10.1089/soro.2017.0076 30024312

[36] Nilsen T, Hermann M, Eriksen CS, Dagfinrud H, Mowinckel P, Kjeken I (2012). Grip force and pinch grip in an adult population: reference values and factors associated with grip force. Scand J Occup Ther.

[37] Kapadia N, Zivanovic V, Verrier M, Popovic M (2012). Toronto Rehabilitation Institute–hand function test: assessment of gross motor function in individuals with spinal cord injury. Top Spinal Cord Inj Rehabil.

[38] Marquez-Chin C, Marquis A, Popovic MR (2016). BCI-triggered functional electrical stimulation therapy for upper limb. Eur J Transl Myol.

[39] Matheus K, Dollar AM (2010). Benchmarking grasping and manipulation: properties of the objects of daily living. Proc - IEEE/RSJ 2010 Int Conf Int Robot Syst (IROS).

