# Supplementary material for: Assisting hand function after spinal cord injury with a fabric-based soft robotic glove
Source: J Neuroeng Rehabil. 2018 Jun 28;15:59. doi: 10.1186/s12984-018-0391-x (PMC6022347; doi:10.1186/s12984-018-0391-x)
Supplement: Supplementary file 1 — Additional views of the fabric-based soft robotic glove. (PDF 430 kb) [file 12984_2018_391_MOESM1_ESM.pdf]

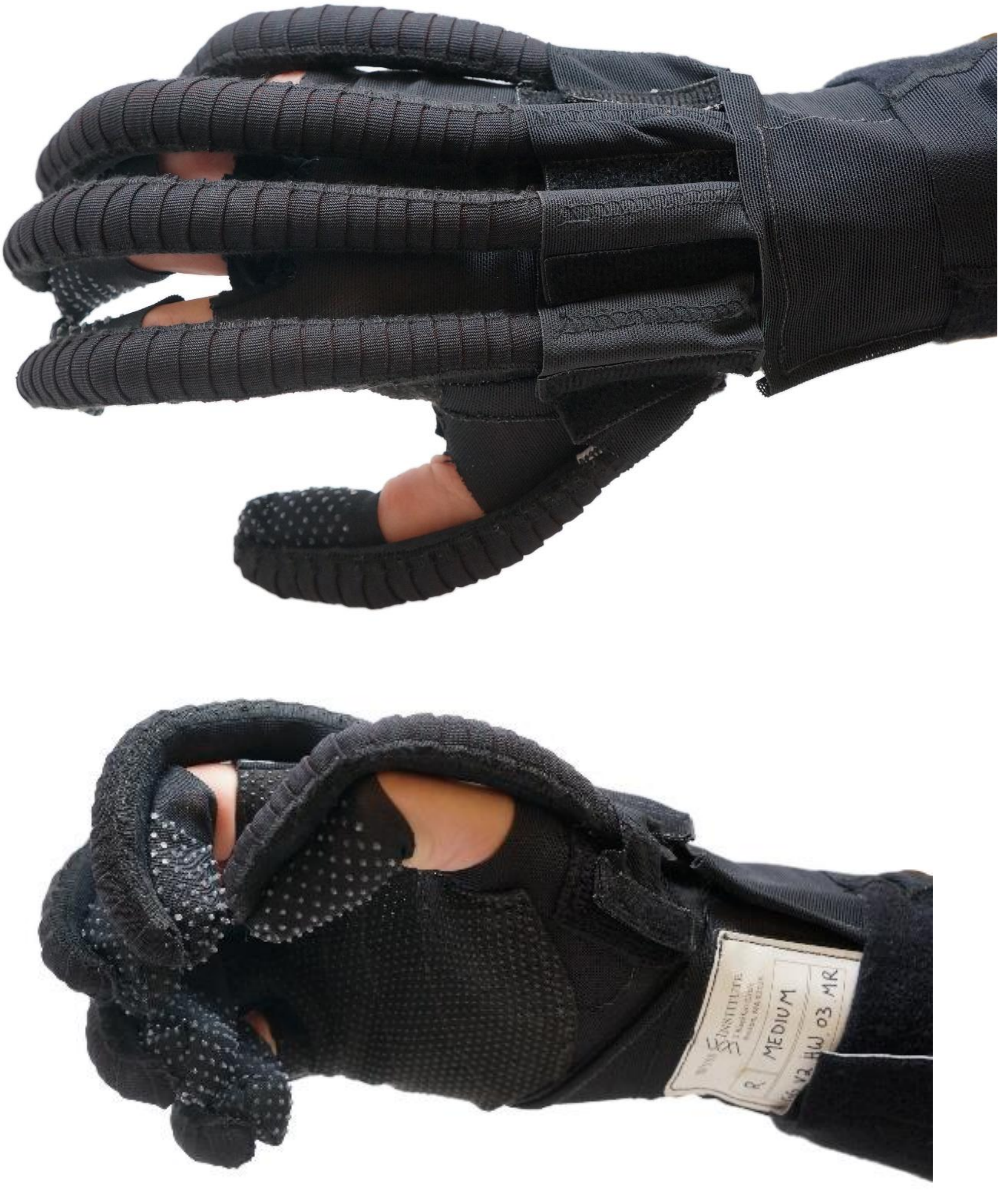

Supplementary Figure 1: Views of the glove during pressurization of the actuators

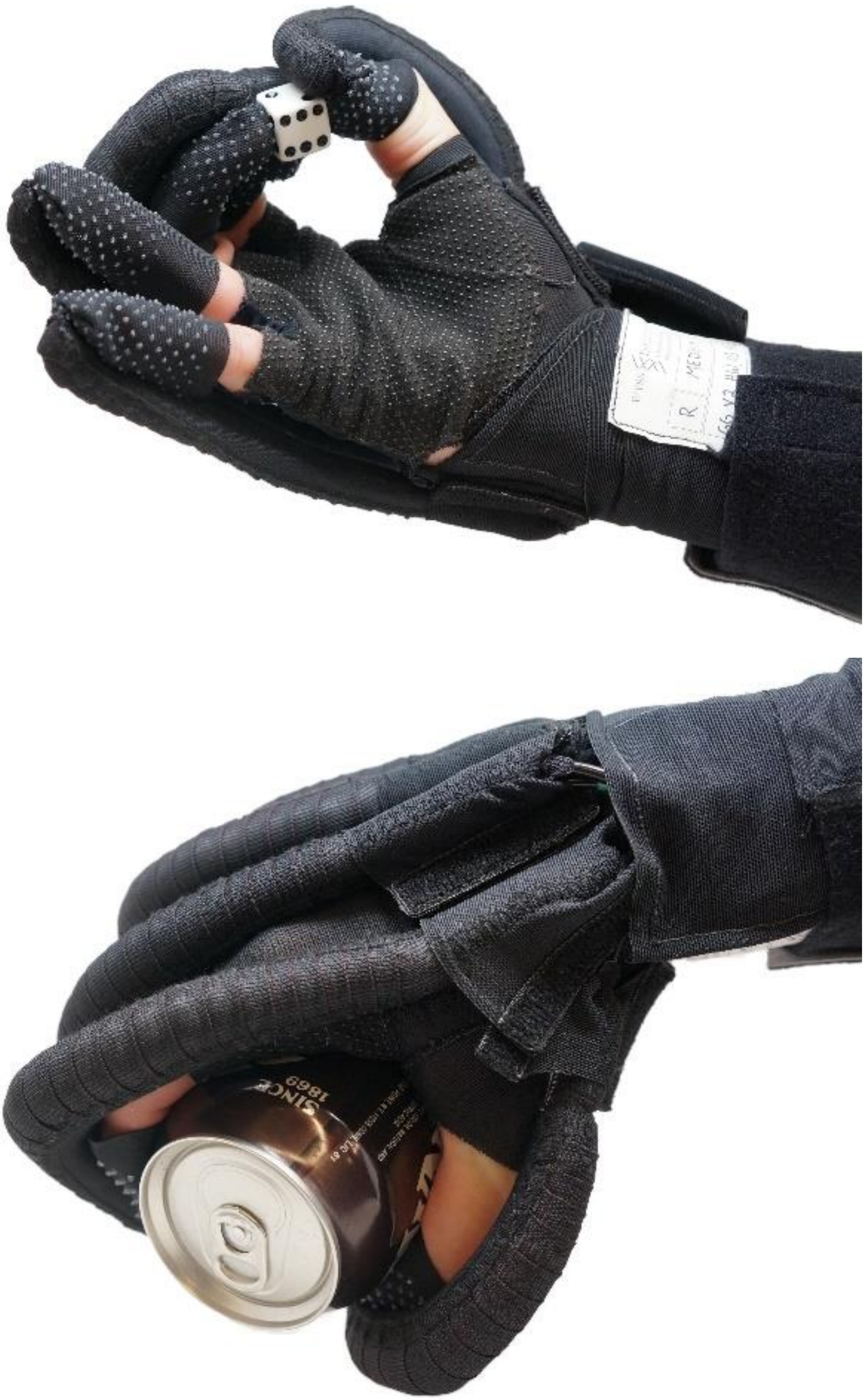

Supplementary Figure 2: Views of the glove during pinch and power grasp

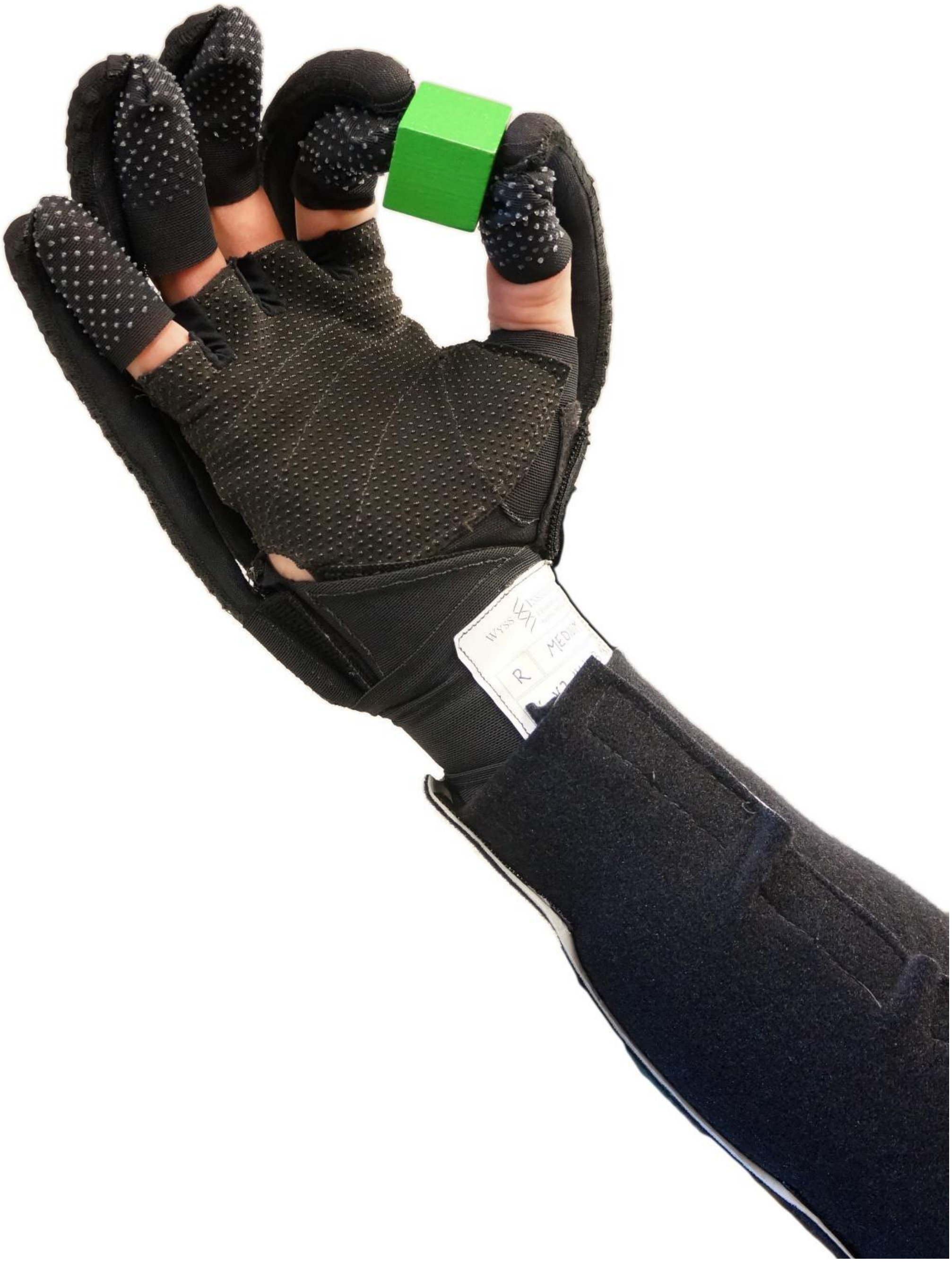

Supplementary Figure 3: Full view of the glove
